# Prevalence of malpractice claims after arthroscopic shoulder surgery: analysis of 69,097 procedures from a national registry in Norway

**DOI:** 10.1186/s13037-023-00378-5

**Published:** 2023-10-18

**Authors:** Ola Midtsaether Lian, Per-Henrik Randsborg, Rune Bruhn Jakobsen, Ida Rashida Khan Bukholm, Tommy Frøseth Aae

**Affiliations:** 1 Department of Orthopaedic Surgery, Helse Møre and Romsdal HT, Kristiansund Hospital, Kristiansund, Norway; 2 Faculty of Medicine and Health Sciences, NTNU, Trondheim, Norway; 3 Department of Orthopaedic Surgery, Akershus University Hospital, Lørenskog, Norway; 4 Institute of Clinical Medicine, Faculty of Medicine, University of Oslo, Oslo, Norway; 5 Department of Health Management and Health Economics, Institute of Health and Society, The Medical Faculty, University of Oslo, Oslo, Norway; 6 The Norwegian System of Patient Injury Compensation, Oslo, Norway; 7 Department of Neuromedicine and Movement Science, Faculty of Medicine and Health Sciences, NTNU, Trondheim, Norway

## Abstract

**Background:**

Systematic analysis of compensation claims following patient injuries is helpful in improving patient safety. The objective of the current study was to assess compensation claims after arthroscopic treatment of rotator cuff ruptures, impingement, and acromioclavicular joint osteoarthritis reported to the Norwegian System of Patient Injury Compensation and evaluate if there was regional variation on the risk of patient injuries leading to an accepted compensation claim.

**Methods:**

Data from the Norwegian System of Patient Injury Compensation and the Norwegian Patient Registry (NPR) from 2008 to 2018 were collected. Demographics (age and sex) and type of claim and reasons for accepted claims were obtained from the Norwegian System of Patient Injury Compensation, while the number of arthroscopic procedures was collected from NPR. The treating institutions were grouped on geography according to Norway’s four regional Health Trusts and private institutions and the effect of geography on the probability of an accepted claim was estimated.

**Results:**

NPR registered 69,097 shoulder arthroscopies during the study period, of which 216 (0.3%) compensation claims were filed for patient injury. A total of 38% of the claims were accepted, representing 0.1% of the arthroscopic procedures. Infection (37.8%) was the most common reason for accepted claim, followed by no surgical indication (15.9%) and wrong surgical technique (12.2%). We found a statistically significantly increased odds ratio for a claim being accepted in both the smallest and largest regional Health Trusts compared to the other regional Health Trusts and private institutions.

**Conclusions:**

Compensation claims due to patient injury following shoulder arthroscopy are rare (0.3% of patients file a claim, of which a third is accepted (0.1% of all shoulder arthroscopy patients)). The most common reason for accepted claim was infection followed by lack of indication.

## Background

Shoulder arthroscopy is one of the most commonly performed orthopedic procedures [1]. The procedure has gained popularity because it shortens hospital stay, is minimally invasive, and the recovery is quicker compared to open surgery [2].

The Norwegian System of Patient Injury Compensation handles all compensation claims following medical treatment in any Norwegian health institution, public or private [3]. This free of charge national compensation system requires three conditions to be fulfilled for a claim to be accepted [3]. Firstly, the patient injury must lead to a financial loss currently set at 10,000 NOK (approximately 900 EUR). Secondly, the injury must have occurred during examination, diagnosis, treatment (or lack thereof) or during follow-up [3]. Finally, the compensation claim must be reported within three years after injury [3].

Previous studies have analyzed compensation claims after hip, knee, and spine surgery [4–7]. Only a few studies have examined compensations claims after shoulder surgery [8–10], but to our knowledge, none has assessed compensation claims following arthroscopic shoulder surgery. The purpose of this study was to assess compensation claims after arthroscopic treatment of rotator cuff ruptures, shoulder impingement and acromioclavicular joint osteoarthritis filed to the Norwegian System of Patient Injury Compensation from 2008 to 2018 and evaluate possible regional effects on the risk of patient injuries ending with an accepted claim.

## Methods

A cross-sectional study was performed in late 2021 in Norway. All patients of any age in Norway who filed a compensation claim to the Norwegian System of Patient Injury Compensation after arthroscopic treatment of rotator cuff ruptures, shoulder impingement and acromioclavicular joint osteoarthritis during the study period (2008–2018) were included.

The Norwegian Patient Registry (NPR) is one of Norway’s national health registries. It is governed by the Norwegian Directorate of Health and was established in 1997. NPR’s primary function is to gather information about patients that are awaiting treatment or under treatment by the specialist health care in Norway.

Data from the Norwegian System of Patient Injury Compensation from 2008 to 2018 and the corresponding volume of procedures were collected from NPR. The patients were identified by electronic searches of the procedure codes in the Norwegian System of Patient Injury Compensation and NPR databases (Table 1). The Norwegian System of Patient Injury Compensation data contained patient demographics, reason for claim, outcome of the claim, treatment institution and procedure performed.

The data from NPR contained the number of the different procedures performed by each of Norway’s four regional Health Trusts or private institutions. Volume per procedure per regional Health Trust was recorded.

### Surgical procedures codes used to search the databases

**Table 1:** The Nordic Medico-Statistical Committee Classification of Surgical Procedures codes used to search the Norwegian System of Patient Injury Compensation and the Norwegian Patient Registry databases

Procedure code	Procedure description
NBA11	Shoulder arthroscopy
NBK12	Resection or excision of clavicula
NBK13	Resection or excision of scapula
NBL49	Suture or reinsertion of tendon in shoulder or upper arm
NBM79	Excision of bursa in shoulder or upper arm

### Statistics

Continuous variables were presented as mean (SD) and categorical variables were described in frequencies. Chi square test was performed to analyze differences in categorical data between the groups. Associations between geographical regions and procedures were assessed with odds ratio.

The statistical significance level was set at 5%. The data was analyzed using Statistical Package for the Social Sciences (SPSS Version 27.0. IBM Corp. Armok NY).

## Results

A total of 69,097 shoulder arthroscopies designated by the relevant procedure codes were performed nationwide from 2008 to 2018. More than 50% of these were acromion resections (51.8%). Clavicle resections and tendon sutures constituted about 20% each (21.8% and 23% respectively). Nearly 2% of the procedures were coded as shoulder arthroscopies alone (unspecified) while the remaining 1% were coded as bursal excision.

Of the four regional Health Trusts, two-thirds of the procedures were performed by the South-Eastern and Western regional Health Trusts (36.4% and 30.6% respectively), followed by the Central regional Health Trust (12.9%) and Northern regional Health Trust (9.6%). Sex and age distribution were not available from NPR.

Within the study period, 216 compensation claims were filed to the Norwegian System of Patient Injury Compensation. The overall likelihood of a compensation claim following shoulder arthroscopy was low, with a ratio of compensation claim per procedure of 0.3%. Among the 216 claims, 82 (38%) were accepted, representing 0.1% of all arthroscopic procedures performed in Norway during the study period.

The majority of compensation claims were filed by men (N = 118, 54.6%) (Table 2).

### Sex distribution of 216 compensation claims

**Table 2:** Sex distribution of 216 compensation claims for patient injury following shoulder arthroscopy in Norway from 2008–2018. n – number

	Accepted claims (n = 82)	Rejected claims (n = 134)	Total (n = 216)
Men, n (%)	49 (59.8)	69 (51.5)	118 (54.6)

**Fig. 1:**
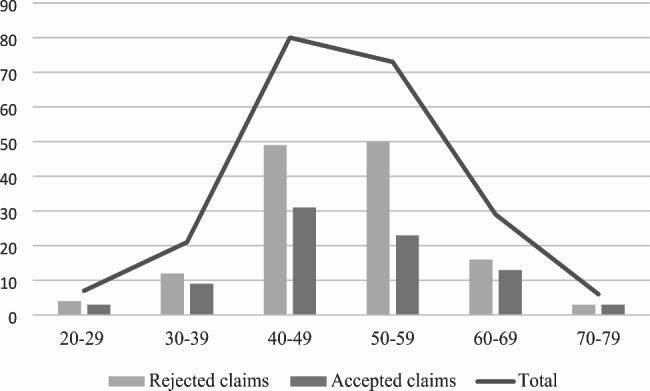
Age distribution of 216 compensation claims for patient injury following shoulder arthroscopy in Norway from 2008–2018

Most compensation claims were filed by patients aged 40 to 60, with few claims registered for patients younger than 30 years old and older than 70 years. (Fig. 1).

### Age distribution of 216 compensation claims

The main reason for accepted claims was infection (37.8%) followed by lack of indication for the surgical procedure (15.9%) and wrong surgical technique (12.2%) (Table 3).

### Reasons for accepted compensation claims

**Table 3:** Reasons for accepted compensation claims after shoulder arthroscopy in Norway from 2008–2018

Reasons for accepted claim	Number	Percent
Infection	31	37.8
No surgical indication	13	15.9
Wrong surgical technique	10	12.2
Postoperative pain	9	11
Delayed treatment	5	6.1
Incomplete perioperative diagnostics	5	6.1
Incomplete preoperative diagnostics	2	2.4
Wrong diagnosis	1	1.2
Operated in wrong shoulder	1	1.2
Reduced postoperative function	1	1.2
Nerve injury	1	1.2
Insufficient follow-up	1	1.2
Positioning injury	1	1.2
Other	1	1.2
Total	82	100

**Fig. 2:**
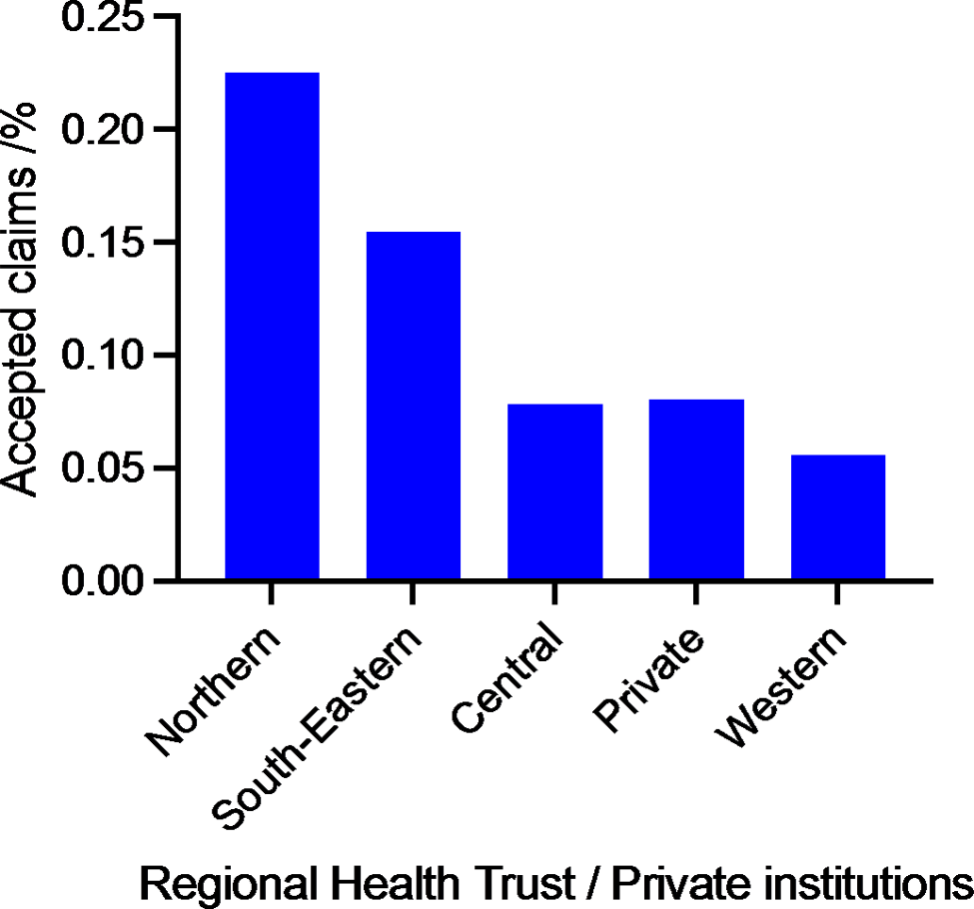
Accepted compensation claims stratified by Norway’s four regional Health Trusts and the private institutions

Among the regional Health Trusts we found a statistically significantly increased odds ratio for an accepted claim per procedure in both the Northern and the South-Eastern regional Health Trusts compared to the other regional Health Trusts (Fig. 2). The private institutions were no better or worse than the public hospitals regarding compensation claims per procedure volume (Fig. 2).

### Accepted compensation claims stratified by institutions

According to procedures, NBA11 – a plain shoulder arthroscopy was identified as more likely to be associated with an accepted compensation claim than the other procedures (p < 0.05) (Fig. 3).

### Percentage of accepted claims according to procedures

**Fig. 3:**
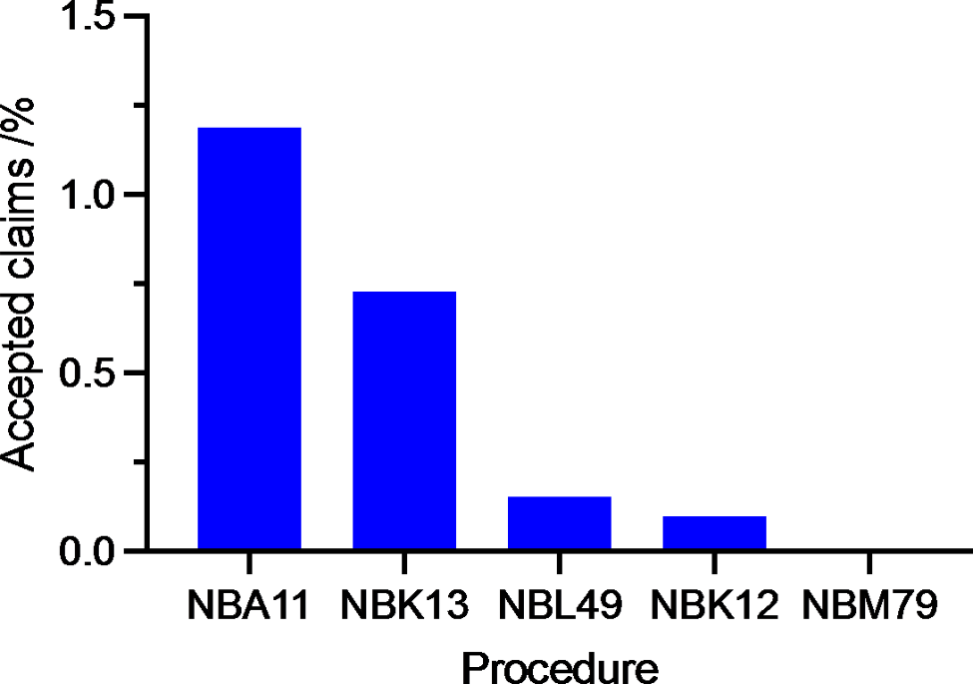
Percentage of accepted claims according to procedures. NBA11 – Shoulder arthroscopy, NBK12 – Resection or excision of clavicula, NBK13 – Resection or excision of scapula, NBL49 – Suture or reinsertion of tendon in shoulder or upper arm, NBM79 – Excision of bursa in shoulder or upper arm (No accepted claims registered)

## Discussion

In this study we find that compensation claims are rare after shoulder arthroscopy. Only 216 claims out of 69,097 shoulder arthroscopies were filed, accounting for 0.3% of all operations. This is lower compared to other elective orthopedic procedures such as hip- and knee arthroplasty (1.9% and 0.9% respectively) [6, 7]. However, these procedures are open surgeries with high expectations and wider complications which may partially explain the difference found in our material. In a retrospective study from Finland, 8 901 compensation claims after various surgical procedures were analyzed [11]. Similar to our findings, they reported an overall claim rate of 6 per 1000 procedures (0.6%) but denoted that “different surgical procedures exhibit varying claims”. Similar to other studies, we found an acceptance rate of compensation claims at nearly 40% [4, 5, 7, 11].

Our data demonstrates that more men than women file a complaint. This is somewhat different to previous findings, where women more often file a compensation claim [6, 7]. Some diagnosis and procedures are more common for men, while others are more common for women. This slightly different gender imbalance may explain why more men than women filed a compensation claim after shoulder arthroscopy compared to hip replacement surgery.

The main reason for an accepted compensation claim was infection (37.8%). This is consistent with previous studies on compensation claims following orthopedic surgery [5–7]. According to regulatory laws governing the Norwegian System of Patient Injury Compensation system, patient claims due to infection are accepted as long as the patient does not have an underlying disorder predisposing for infection. These claims are still evaluated for patient injury, but acceptance is granted independently of this investigation if no predisposing factors are identified.

No indication for surgery was the second most common reason for accepted compensation claims (15.9%). This demonstrates the importance of accuracy in diagnosis and indication for surgery and is a reminder that non-operative treatment often is a valid solution. There is an increased focus on low-value care, referring to the use of health care service for which harms or costs outshine the therapeutic benefits [12, 13]. The Choosing wisely campaign, originating from the United States, have become an international strategy to reduce wasteful healthcare [14]. In arthroscopic shoulder surgery, several studies have demonstrated that less equals more [15, 16]. Schrøder et al. showed that neither labral repair nor biceps tenodesis for superior labrum anterior posterior lesion of the shoulder is superior to sham surgery [15]. Our study indicates that experts at the Norwegian System of Patient Injury Compensation have caught on to the change in indication for some arthroscopic procedures, and that publications such as this one may help direct attention to low value care in orthopaedics.

The Norwegian System of Patient Injury Compensation experts concluded that ten patients had suffered a patient injury due to “wrong surgical technique”. This included insufficient bone removal in shoulder impingement procedures, loose/misplaced anchors after rotator cuff surgery and insufficient surgical capsulotomy before frozen shoulder mobilization. No common denominator was found among these patients, but is should be mentioned that only one Norwegian System of Patient Injury Compensation expert assesses a compensation claim making the assessment susceptible to subjectivity. It is important to point out that the Norwegian System of Patient Injury Compensation experts had the luxury of hindsight when reviewing the claims. A clearer guideline for the experts or multiple reviewers is desirable.

Pain was a common reason for filing a complaint, as 95 patients (43%) filed a complaint due to postoperative pain. However, pain is not a common cause for accepted claims as only 9 of 95 patients (9.5%) received compensation due to persistent postoperative pain. This finding is consistent with other studies [5, 8].

One patient suffered a cerebral stroke after an elective subacromial decompression. Acceptance was granted due to both faulty indication of the surgery and the consequences for the patient which were described as disproportional to the potential benefits of the surgery.

One patient was operated in the wrong shoulder. This occurred in 2013. Although various safe surgery checklist was implemented in Norway from 2012 to reduce and prevent treatment errors [17], this unfortunate accident demonstrates the importance of vigilance in all health care aspects.

A total of 7 patients had their compensation claims accepted due to inadequate diagnostics. This is a reminder of the importance of taking the time and performing the necessary tests required to arrive at the correct diagnosis before committing to a treatment strategy, especially if this involves surgery. The time interval from diagnosis to surgical treatment was considered too long for 5 patients (6.1%). The reparability of a rotator cuff rupture decreases in time. Within months, fatty infiltration and atrophy limit the mobility of the rotator cuff causing an eventual irreversible retraction of the tendon [18]. Particularly in the presence of a traumatic rupture, time is of the essence.

In a study involving malpractice claims against shoulder surgeons, Lynch et al. identified only 45 malpractice claims over a 28-year period [8]. This study included arthroscopic and open surgery and reported that rotator cuff repairs had the greatest total number of litigations, whereas manipulation under anaesthesia had the highest probability to be litigated. This is somewhat different to our findings. We found NBA11 (shoulder arthroscopy) to be the most likely procedure to lead to a patient injury and an accepted claim (p < 0,05). This procedure code is often used as a primary code in more complex arthroscopies (i.e. shoulder stabilization, capsulotomies) as well as minor surgeries (i.e. diagnostic procedures, removal of corpora libra). The surgical diversity differs more within this group than in the other groups. This might at least in part explain why this group has more acceptances than the other groups.

A puzzling finding in our study is that there is a complex relationship between hospital geography and accepted compensation claims following shoulder arthroscopy. We found a significant increased odds ratio for an accepted claim per procedure in both the Northern and the South-Eastern regional Health Trusts compared to the other regional Health Trusts and the private institutions. The Northern regional Health Trust is the smallest regional Health Trust in Norway both in terms of catchment population and the number of shoulder arthroscopies, while the South-Eastern regional Health Trust is the largest and performed most arthroscopies. It is not unexpected that the regional Health Trust with the fewest surgeries had a higher risk for accepted compensation claims, as this is consistent with previous findings that lower volume is proportional to accepted compensation claims [5, 7]. The fact that we found an increased risk of accepted compensation claim also in the highest volume regional Health Trust is somewhat surprising. However, two of Norway’s most high-profile shoulder centres that treat complex and difficult shoulder injuries are located in this region, which may explain this finding.

There are some limitations to our study. We lacked clinical information on sex and age from NPR, and the diagnosis codes are broad and cover many different procedures, which makes it difficult to find subgroups of interventions that might have a particularly high risk of patient injury. We have only included surgical treatment options for common shoulder disorders and excluded non-surgically treated patients. Our data is from a single country, with a compensation claim system that differs from other countries, which may affect the generalizability of our study. However, identifying areas of improvement in health care should be of global interest. It is likely that patient injuries have occurred during the study period that was not reported to the Norwegian System of Patient Injury Compensation. The threshold for filing a complaint is highly individual. It is likely that a study on compensation claims is underestimating the prevalence of patient injury since not all patient injuries are reported to the Norwegian System of Patient Injury Compensation.

## Conclusion

Compensation claims due to patient injury following shoulder arthroscopy are rare (0.3% of patients file a claim, of which a third is accepted (0.1% of all shoulder arthroscopy patients)). The most common reason for accepted claim was infection followed by lack of indication.

## Data Availability

Data is available upon application and a compulsory fee to NPR and the Norwegian System of Patient Injury Compensation.

## Abbreviations

NPR: Norwegian Patient Registry

## References

[1] Judge A, Murphy RJ, Maxwell R, Arden NK, Carr AJ (2014). Temporal trends and geographical variation in the use of subacromial decompression and rotator cuff repair of the shoulder in England. Bone Joint J.

[2] Baker DK, Perez JL, Watson SL, McGwin G, Brabston EW, Hudson PW, Ponce BA (2017). Arthroscopic Versus Open Rotator Cuff Repair: which has a better complication and 30-Day Readmission Profile?. Arthroscopy.

[3] The history of. the patient injury compensation scheme https://www.npe.no/en/About-NPE/Organisation/The-history-of-the-patient-injurycompensation-scheme/.

[4] Randsborg PH, Bukholm IRK, Jakobsen RB (2018). Compensation after treatment for anterior cruciate ligament injuries: a review of compensation claims in Norway from 2005 to 2015. Knee Surg Sports Traumatol Arthrosc.

[5] Aae TF, Lian ØB, Årøen A, Engebretsen L, Randsborg PH (2020). Compensation claims after knee cartilage Surgery is rare. A registry-based study from Scandinavia from 2010 to 2015. BMC Musculoskelet Disord.

[6] Randsborg PH, Aae TF, Bukholm IRK, Fenstad AM, Furnes O, Jakobsen RB (2021). Compensation claims after knee arthroplasty Surgery in Norway 2008–2018. Acta Orthop.

[7] Aae TF, Jakobsen RB, Bukholm IRK, Fenstad AM, Furnes O, Randsborg PH (2021). Compensation claims after hip arthroplasty Surgery in Norway 2008–2018. Acta Orthop.

[8] Lynch JC, Radack TM, Stenson JF, Riebesell SA, Austin LS (2022). Malpractice against shoulder surgeons: what the data say. J Shoulder Elbow Surg.

[9] Sharma A, Whitlock KG, Gage MJ, Lassiter TE, Anakwenze OA, Klifto CS (2021). Malpractice trends in shoulder and elbow Surgery. J Shoulder Elbow Surg.

[10] Shin JJ, Popchak AJ, Musahl V, Irrgang JJ, Lin A (2018). Complications after arthroscopic shoulder Surgery: a review of the American Board of Orthopaedic Surgery Database. J Am Acad Orthop Surg Glob Res Rev.

[11] Welling M, Takala A (2023). Patterns of malpractice claims and compensation after surgical procedures: a retrospective analysis of 8,901 claims from the Finnish patient insurance registry. Patient Saf Surg.

[12] Oakes AH, Radomski TR (2021). Reducing low-value care and improving Health Care Value. JAMA.

[13] McCormack RG, Hutchinson MR (2017). Rocking the shoulder surgeon’s world. Br J Sports Med.

[14] Born KB, Levinson W (2019). Choosing wisely campaigns globally: a shared approach to tackling the problem of overuse in healthcare. J Gen Fam Med.

[15] Schrøder CP, Skare Ø, Reikerås O, Mowinckel P, Brox JI (2017). Sham Surgery versus labral repair or biceps tenodesis for type II SLAP lesions of the shoulder: a three-armed randomised clinical trial. Br J Sports Med.

[16] Kukkonen J, Joukainen A, Lehtinen J, Mattila KT, Tuominen EK, Kauko T, Äärimaa V (2015). Treatment of nontraumatic rotator cuff tears: a randomized controlled trial with two years of Clinical and Imaging follow-up. J Bone Joint Surg Am.

[17] Ahlberg J, Pukk-Härenstam K. [Safe Surgery saves lives – 10 years of Swedish experience]. Lakartidningen 2018, 115. 29917170

[18] Novi M, Kumar A, Paladini P, Porcellini G, Merolla G (2018). Irreparable rotator cuff tears: challenges and solutions. Orthop Res Rev.

